# Global consumption and international trade in deforestation-associated commodities could influence malaria risk

**DOI:** 10.1038/s41467-020-14954-1

**Published:** 2020-03-09

**Authors:** Leonardo Suveges Moreira Chaves, Jacob Fry, Arunima Malik, Arne Geschke, Maria Anice Mureb Sallum, Manfred Lenzen

**Affiliations:** 1 Departamento de Epidemiologia, Faculdade de Saúde Pública, Universidade de São Paulo, São Paulo, SP Brazil; 2 ISA, School of Physics A28, The University of Sydney, Sydney, NSW 2006 Australia; 3 Discipline of Accounting, The University of Sydney Business School, The University of Sydney, Sydney, NSW 2006 Australia

**Keywords:** Ecological epidemiology, Environmental economics, Risk factors

## Abstract

Deforestation can increase the transmission of malaria. Here, we build upon the existing link between malaria risk and deforestation by investigating how the global demand for commodities that increase deforestation can also increase malaria risk. We use a database of trade relationships to link the consumption of deforestation-implicated commodities in developed countries to estimates of country-level malaria risk in developing countries. We estimate that about 20% of the malaria risk in deforestation hotspots is driven by the international trade of deforestation-implicated export commodities, such as timber, wood products, tobacco, cocoa, coffee and cotton. By linking malaria risk to final consumers of commodities, we contribute information to support demand-side policy measures to complement existing malaria control interventions, with co-benefits for reducing deforestation and forest disturbance.

## Abstract (web summary)

Because many primary commodities cause deforestation and deforestation can increase malaria transmission, international trade can thus indirectly influence malaria risk. Here the authors use trade databases for commodites associated with deforestation to demonstrate that consumption of such commodities in developed nations could increase malaria risk in developing nations.

## Introduction

Malaria is a tropical and subtropical disease caused by any one of five species of the Apicomplexa parasite: *P*. *falciparum*, *P*. *vivax*, *P. malariae*, *P*. *ovale*, and *P*. *knowlesi*. Even though malaria incidence has decreased globally, there have been worrying developments in recent years^1^. The persistence of social vulnerability and poverty, as well as continuous changes in natural landscapes for economic development and population growth, provide favorable environments for malaria transmission, ultimately translating into high incidence. In 2018, there were an estimated 228 million cases of malaria worldwide, mostly in developing countries, resulting in 405,000 deaths, 67% of which were children under 5 years old, and 94% of child <5 years old deaths were in Sub-Saharan Africa^2^. Despite recent advances in the development of malaria vaccines as well as drug treatments and rapid diagnostic tests for *Plasmodium* detection^3^, maintaining the upper hand in the fight against malaria remains a difficult and expensive task for endemic countries^2^. The World Health Organization (WHO) has emphasized the need for a strategic integrated global vector control program^4^, under the United Nations Sustainable Development Agenda (UNSDA)^5^.

**Fig. 1:**
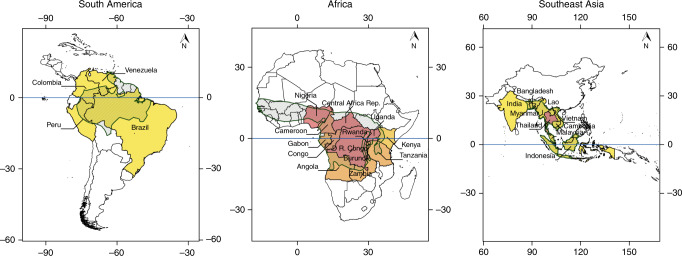
Malaria incidence by country in the world’s three largest rainforests, overlaid with deforestation frontier. Shaded countries: Populations at risk (cases per 1000 population at risk from 2000–2015) (yellow <140, orange 140–200, light brown 200–270, dark brown >270). Hatched areas: deforestation frontiers. Further detail in Methods. Software used: QGIS version 2.8 without any changes. QGIS is licensed under Creative Commons Attribution-ShareAlike 3.0 licence (CC BY-SA) (https://creativecommons.org/licenses/by-sa/3.0/).

More than 90% of all human malaria occurs in the world’s three largest tropical rainforest biomes, and adjacency: the Amazon Basin, the Congo Basin and the Greater Mekong^2^. Various studies have shown that the incidence of human malaria and the abundance and distribution of its primary mosquito vectors, *Anopheles gambiae*, *Anopheles funestus*, *Anopheles dirus*, *Anopheles minimus,* and *Nyssorhynchus darlingi*, are associated with deforestation (Fig. 1), exploitation of natural resources, human migration, changes in land occupation and land use (see Supplementary Note 1 for a detailed review of literature showing evidence of the link between malaria and deforestation).

The inter-relationship between malaria parasites and human hosts is associated with adaptive mutations in the human genome. A recent study that focused on the evolution of human populations that were highly exposed to malaria provides an additional argument in favour of the association between deforestation and malaria^6^.

In particular, for the case of Indonesia, Garg^7^ demonstrated that one standard deviation decline in forest cover measured in each pixel, as unit of analysis, increases the likelihood of malaria outbreaks by 1.85 or 10 percentage points after controlling for other cofactors, such as migration, land use and implementation of anti-malarial control programs. In Nigeria, Berazneva and Byker found a similar association between changes in forest cover and malaria in children under 5 years old^8^. An interdisciplinary, cross-national study employed structural equations models to provide robust evidence of the interconnections and pathways among rural population growth, agricultural specialization, forest loss, and malaria prevalence in 67 countries where malaria is endemic^9^. Recently, MacDonald and Mordecai^10^ tested the hypothesis of a bidirectional socioecological feedback between deforestation and malaria using a dataset encompassing 795 Amazonian municipalities across 13 years. Their results estimated that a 10% increase in forest area cleared can lead to a 3.3% increase in malaria incidence, and a 1% increase in malaria incidence could decrease 1.4% of forest area cleared. A positive correlation between the number of forest patches affected by deforestation less than 5 km^2^ and the incidence of malaria was found in areas across the Brazilian Amazon: each kilometer square of deforestation resulted in 27 new malaria cases^11^. In the westernmost municipality in the Brazilian Amazon, statistical associations between malaria incidence and cumulative percentage of land deforested were found using univariate and multivariate general additive negative binomial models adjusted for spatial trends, access to treatment, and health district size, where a 4.3% increase in deforestation was associated with a 48% increase in the incidence of malaria^12^.

Various studies related to vector ecology have focused on entomological aspects linked to deforestation and malaria in East African^13–16^; Southeast Asia^17,18^; ecological frontiers in endemic regions^19^; agricultural frontiers^20^, tropical America^21–25^; among others (Supplementary Note 1). Forest cover loss allows more sunlight to reach the soil, leading to an increase in temperature of the larval habitats and the formation of puddles with neutral pH, thus favoring larval development, as observed in *Ny*. *darlingi*^26^. Deforestation has been shown to reduce the larva-to-adult development time and increase adult survivorship^14,26–29^, which in turn can further increase malaria risk. A recent study explained how deforestation can directly shape local Anophelinae community composition and possible scenarios of malaria transmission. In the endemic region of Urabá, Antioquia, northwest Colombia, *Nyssorhynchus nuneztovari* is the most abundant and dominant malaria vector species in deforested landscapes with grass, shrub, and bare soil land cover. In contrast, *Ny*. *darlingi* is favored in landscapes with small deforested patches^25^. In addition, deforestation decreases biodiversity, causing a reduction in the abundance of species that prey on Anophelinae larvae and adults, leading to an increase in the abundance of vectors^30^. Therefore, irrespective of region and species, anthropogenic disturbance in natural forest landscapes can lead to changes in mosquito communities, causing an increase in the abundance of vector species^24,26,31^, and subsequently the risk of human exposure to vectors and thus to malaria, in areas where the landscape is conducive to vector–human contact^32,33^. These findings can be largely applied to other tropical and subtropical malaria endemic countries because deforestation disrupts environmental conditions, thus exacerbating ecological conditions, which favours species that are vectors of malaria.

Natural resource exploitation linked to deforestation in the Amazon, Congo Basin, and Greater Mekong is expedited by the production of primary commodities such as timber, soybean, beef, palm oil, tobacco, cocoa, coffee, and cotton^34^, which are driven by demand in developed countries^35^. This exploitation can have a positive impact on local economic growth but can also dramatically degrade tropical forests^34^ and threaten animal species^36^. The demand for consumer goods in developed countries and subsequent primary commodity production are driving changes in tropical forest landscapes that, in turn, increase malaria risk. The communities likely to be at greatest risk are those exposed to mosquito vectors and at the same time facing landscape transformation, and ironically, those are the people who benefit the least economically^37^. In this study, we pursue this line of investigation, by linking malaria incidence in developing countries directly to products demanded by distant consumers. We achieve this by quantitatively relating malaria incidence first with deforestation, then to primary commodity production, which we then connect to the global supply-chain network and ultimately to worldwide consumer demand. The final step is accomplished by coupling a highly detailed and large international trade database^38^ with an established and widely used analytical technique—multi-regional input–output (MRIO) analysis^39^.

The aims of this study are: (1) to gain an understanding of how export-oriented production leads to exploitation of natural forest environments in malaria endemic countries, (2) to reveal connections facilitated by the international trade network between consumers and forestry producers in malaria-prone countries; and based on this understanding (3) to identify countries that face significant malaria risk from global consumption and international trade. This work goes beyond simple incidence mapping and correlations, in that it unveils a global supply-chain network that links malaria occurring in specific locations because of deforestation with globally dispersed consumption.

Here we show that about 20% of the malaria risk in deforestation hotspots is driven by international trade. We further show that in 2015, about 110 million people were at risk of contracting malaria due to deforestation. We conclude by suggesting the need for implementation of initiatives aimed at reducing deforestation and forest disturbance.

We hope that our study provides information about potential demand-side measures for mitigating malaria, focusing on the role of international trade and export dependence for accelerated deforestation and therefore malaria risk. Innovative demand-side measures can complement existing malaria control interventions such as insecticide-treated mosquito nets (ITN) and artemisinin-based combination therapies (ACT), in that they can be effective in regulating and curtailing demand for internationally traded malaria-implicated commodities such as timber, soy, beef, and palm oil. Some demand-side measures already exist for mitigating biodiversity threats^36^, child labour^40^, and global inequality^41^.

## Results

### Overall modelling approach

In order to connect malaria risk with global consumption we apply Leontief’s method^39^ (See Methods for more detail) to analyze a global multi-region input–output (MRIO) database, tracing commodities that were initially obtained as a result of deforestation in tropical forests, then transformed throughout a complex network of international processing chains, and finally delivered to their ultimate destinations in developed-country households. This method has been applied previously to assess the effect of international trade on biodiversity loss^36^, transboundary health impacts of global air pollution^42^, and many other environmental and social indicators^43^. Here, we compute a malaria footprint tensor $F_{ij}^{rst} = q_i^rL_{ij}^{rs}y_j^{st}$, where $q_i^r$ is a measure of malaria risk during the production of (deforestation-linked) commodity *i* in (usually tropical) country *r*; $L_{ij}^{rs}$ is the Leontief inverse of the global economy, describing the transformation of malaria-implicated primary commodities into consumer items *j* manufactured for sale in countries *s*; and $y_j^{st}$ describes the final consumption of these items by households in countries *t*.

Malaria footprints cover entire global supply-chain networks. For example, let $q_i^r = \frac{{Q_i^r}}{{x_I}}$ represent the number of malaria cases $Q_i^r$ in *r* = Brazil as a consequence of deforestation to produce an annual output of *x*_*i*_ dollars of *i* = soybeans. Let $A_{il}^{ru}A_{lm}^{uv}A_{mn}^{vw}A_{nj}^{ws} \in L_{ij}^{rs}$ represent an international supply chain, with $A_{{{il}}}^{{{ru}}}$ dollars of Brazilian soybeans exported to *u* = Argentina for processing into *l* = soybean meal, $A_{{{lm}}}^{{{uv}}}$ dollars of Argentinian soybean meal exported to *v* = Vietnam for processing into *m* = animal feed pellets, $A_{{{mn}}}^{{{vw}}}$ dollars of Vietnamese animal feed pellets exported to *w* = Thailand for feeding *n* = chicken, and finally $A_{{{nj}}}^{{{ws}}}$ dollars of Thai poultry meat exported to *s* = Japan’s *j* = restaurant sector. $A_{il}^{ru}A_{lm}^{uv}A_{mn}^{vw}A_{nj}^{ws}$ is a 4-node international supply chain, and only one out of a large number of supply chains connecting Brazilian soybean production with Japanese restaurants. Finally, let $\it y_j^{{\mathrm{st}}}$ be the consumption of a Japanese restaurant meal containing Thai chicken by a tourist from *t* = Germany. Then, $q_i^rA_{il}^{ru}A_{lm}^{uv}A_{mn}^{vw}A_{nj}^{ws}y_j^{st}$ represents the malaria risk transferred by this consumption decision to people living in Brazil’s expanding soybean-growing frontier areas. Finally, $F_{ij}^{rst}$ represents the malaria risk transferred by the combination of all possible supply chain combinations that link countries *r* and *t* via *s*, and involve primary and final commodities *i* and *j*. The number of such combinations is very high: for an MRIO system distinguishing 100 countries and 100 commodities there would be 10,000 1-node supply chains, 100 million 2-node chains, one trillion 3-node chains, and 10^4*n*^ *n*-node chains in general. Therefore, evaluating the tensor $F_{{\mathrm{ij}}}^{{\mathrm{rst}}}$ requires high-performance computation.

The malaria footprint tensor $F_{ij}^{rst}$ can be collapsed into a number of simplified measures. For example, $\mathop{\sum}\limits_{sij} F_{ij}^{rst} = :F_{\bullet\bullet}^{r\bullet t}$ represents the bilateral malaria footprint in *r* caused by consumption in *t*, no matter through which commodities or supply chains. Net malaria risk trade of country *r* is defined as malaria risk exports minus imports $F_{\bullet\bullet}^{r\bullet\bullet}-F_{\bullet\bullet}^{\bullet\bullet r}$, with $F_{\bullet\bullet}^{r\bullet\bullet}\, <\, F_{\bullet\bullet}^{\bullet\bullet r}$ for net importers, and $F_{\bullet\bullet}^{r\bullet\bullet}\,> \, F_{\bullet\bullet}^{\bullet\bullet r}$ for net exporters.

### Malaria footprint

**Fig. 2:**
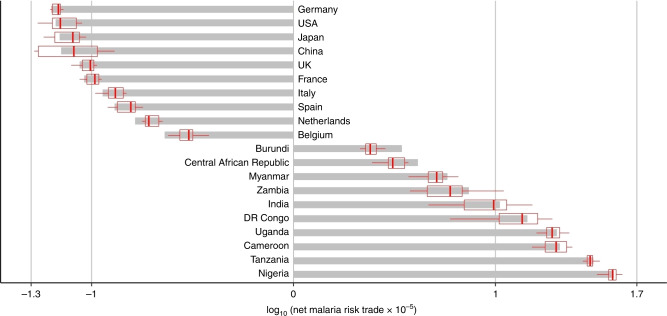
Net malaria risk trade, negative for net importers, and positive for net exporters. The grey bars and the red bold line represent the median and mean, the red box the standard deviation, and the horizontal red thin lines the minimum and maximum of net malaria risk trade over the 2000–2015 period.

Within the confines of the available data and given qualifications of our approach, we find that about 20% of the malaria risk in the world’s deforestation hotspots may be associated with the international trade of deforestation-implicated export cash crops such as timber, cocoa and coffee. These exports are then further processed, and ultimately bound for consumption of affluent consumers in developed countries. The remaining 80% are due to domestic deforestation-linked consumption, for example firewood collection, subsistence or smallholder agriculture for own use^44^. About 10% of malaria risk appears to be linked to just ten countries (net importers in Fig. 2 and Supplementary Table 1), where the demand for certain products could be exacerbating malaria risk for 10.7 million people in low-income, mostly African countries (net exporters in Fig. 2).

**Fig. 3:**
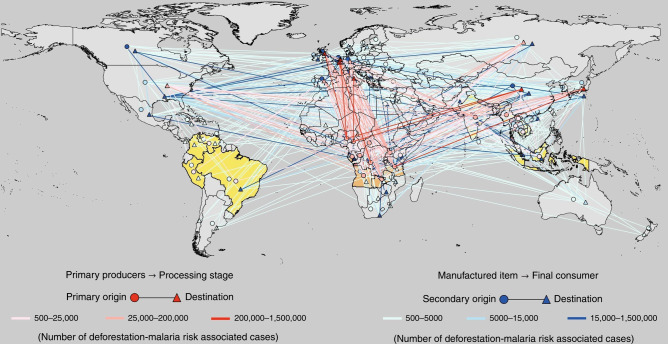
International trade routes of malaria risk associated with traded commodities. Countries with populations at risk are shaded as in Fig. 1. Trade relationships are color-coded, with risk categories falling into three range brackets. A bracket [500-25,000] means that the respective shade of red is used for lines representing the estimated number malaria risk cases. We distinguish (**a**) primary trade routes (red) that originate in countries where deforestation causes malaria risk, and end in countries where commodities are manufactured and assembled for final sale, followed by (**b**) secondary trade routes (blue) that end in countries where commodities are finally consumed by households. Software used: QGIS version 2.8 without any changes. QGIS is licensed under Creative Commons Attribution-ShareAlike 3.0 licence (CC BY-SA) (https://creativecommons.org/licenses/by-sa/3.0/).

Nigeria suffers the highest risk (5.98 million cases in 2015), with demonstrated links to deforestation^8,45^, which in turn is partly caused by the export of timber to China^46^ worth $332 m in 2015^47^, cocoa beans^48^ to the Netherlands ($334 m; all 2015 values from here on), Germany ($72 m), as well as Belgium, France, Spain and Italy ($35 m), and charcoal to Europe ($35 m, Fig. 3 red arrows). Next, in Tanzania, exports of cash crops^49^ such as raw tobacco^50^ to Europe and Asia ($344 m, $96 m to Belgium alone), and raw cotton ($41 m; mainly to South and Southeast Asia), as well as sawn wood to India ($20 m), are some of the contributors to 5.66 million people at deforestation-linked malaria risk^51^ in 2015. Similarly, malaria (5.49 million risk cases) has been linked to deforestation in Uganda^52^, which in turn is potentially driven by exports of raw coffee^53^ to Italy ($88 m), Germany ($63 m), Belgium ($40 m) and USA and Spain (both $21 m), and to a lesser extent raw cotton (South and Southeast Asia; $15 m). Finally, deforestation-linked malaria in Cameroon^54–56^ (5.49 million risk cases) can be connected to cocoa exports^57^ to the Netherlands ($300 m), Spain, Belgium, France and Germany (together $79 m), rough wood to China ($175 m), and sawn timber ($440 m, Belgium, China, Italy, the USA, and many other destinations). The remaining tropical areas can be similarly linked to distant consumers^58^, mainly through exports of sawn and rough timber from the DR Congo, Zambia, Myanmar, Central African Republic and Angola. Their main trading partner is China, whose imports of rough logs (>$500 m) and sawn timber (>$70 m) from the region peaked in 2014. We do not find beef and palm oil in top-ranking trade links, because these commodities are predominantly traded out of countries with a relatively low malaria incidence, such as Brazil and Malaysia.

The trade connections described so far are relatively simple as they consist of only one pair of trading countries and one commodity. However, potential malaria-implicated supply chains can be more complex. In an upstream direction, we find that in the net exporters listed in Fig. 1, processing of tobacco, coffee, cotton, and cocoa beans often consumes large amounts of domestic fuelwood and polewood, which indirectly connects these commodities with deforestation and malaria risk. In a downstream direction (Fig. 3, blue arrows), we find multi-step international trade routes tracing Ugandan and Tanzanian tea^59^ first to Kenya’s Mombasa Tea Auction, from which it then gets shipped to the UK ($157 m). Similarly, Uganda exports tobacco to Kenya ($45 m) for processing and re-export. These circumstances mean that despite Kenya being a country at malaria risk, it is a net importer (−334.821 risk cases). Further down the supply chain, malaria-implicated commodities become transformed into consumer items, and dispersed to households across the globe, often associated with a significant added value and revenue. For example, in 2015, about $6b out of $12b of raw tobacco was exported out of malaria-affected countries, but $20b out of $29b of processed tobacco was finally sold out of Europe and North America^47^. China sources African timber predominantly as rough logs, and adds significant value as it turns these into a plethora of wood products such as furniture^60^, flooring^61^, joinery, and plywood^62^ for export to Japan, Europe and the USA. The Netherlands and Belgium turn annual imports of almost exclusively African cocoa beans worth about $700 m into about $4.6b of chocolates exports, mostly to other European countries. Calculated on a per-consumer basis, Dutch and Belgians potentially cause the world’s highest malaria footprints (often facilitated by cocoa exports) at about 31.25 risk cases per 1000 inhabitants, followed by Swiss and Germans (22.3/1000 risk cases; cocoa and tobacco), the UK (17.85; tea), French, Spaniards, and Italians (13.39; cocoa and coffee), Japanese (8.92; wood products), and US Americans (4.46; various products).

An interesting bird’s-eye view of the supply-chain structure of malaria risk can be gained from a world map (Fig. 3). Primary producers of malaria-implicated raw commodities such as cocoa, tea, coffee, tobacco, and timber, mainly in tropical Africa, but also in India and Myanmar (red circles), ship these for further processing and value-adding to intermediate producers of numerous commodities such as chocolates, cigarettes, furniture, flooring, joinery, and plywood, mainly in China, Japan, and Western Europe (red triangles). From there (blue circles), these commodities reach final consumers in the Americas, Europe, Russia, China, Japan, South East Asia, Australia, and New Zealand (blue triangles). In contrast to primary trade of raw products, which proceeds along a limited number of concentrated trade routes originating from mostly low-income malaria-affected exporters (red), secondary trade disperses manufactured products amongst numerous smaller trade links involving mostly high-income producers and consumers (blue). Interestingly, a striking feature in Fig. 3 is the prominence of primary trade routes between European nations such as the UK, France, Belgium, and Germany and their former African colonies.

**Fig. 4:**
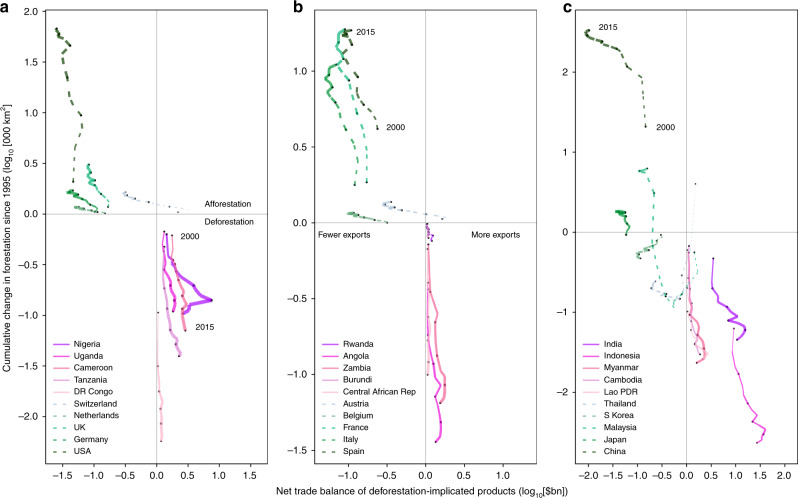
Trends in forestation, trade in deforestation-implicated products, and post-2000 malaria footprints. Panels (**a**), (**b**) and (**c**) each show a collection of net exporting (magenta) and net importing (green) countries. Axes are logarithmic, with negative values representing deforestation (for example −1 equals a cumulative 10,000 km² forest loss since 1995) and product import (for example −2 equals a US$100 bn import). Plotted points represent the years between 2000 and 2015 in 3-year intervals where line thickness is proportional to net malaria risk trade, and net exporters represented by a solid line, net importers by a dashed line. The time series proceeds from the origin outwards and also with increasing line thickness. As time progresses, richer countries import more deforestation-implicated products which causes deforestation in poorer countries, while at the same time preserving more of their own forest.

Developed countries have been continuously outsourcing forest-intensive production to developing countries whilst preserving their own resources and nature. An analysis of annual time series data between 2000 and 2015 reveals that trends of international trade of deforestation-implicated products as well as deforestation are increasing (Fig. 4). First, the trajectories of all countries shown in Fig. 4 proceed from the origin outwards. This is because export commodity crop markets are expanding, intensifying deforestation in malaria-prone regions^63^. Trade statistics show that especially the international trade of rough logs, sawn wood, raw cocoa, coffee, tea, tobacco, and cotton has markedly increased over the analysis period. These trends are responsible for continuing deforestation (cumulative forest losses exceeding 10,000 km² in Nigeria, Uganda, Cameroon and India, and exceeding 100,000 km² in DR Congo and Indonesia). Whilst increasing commodity trade drove deforestation in much of tropical Africa and also South East Asia, many developed countries that import deforestation-implicated products were able to increase their own forest cover^64^.

## Discussion

Although there has been a reduction in global deaths from malaria since 2000^2^, malaria incidences have increased in the past few years, requiring a revision of the political, policy, operational, and financial approaches^65^. The Global Health Group and the *Lancet* Commission devised an initiative to achieve malaria eradication^66^, where the question “How will global megatrends impede malaria eradication?” was raised. In this study, we contribute a response by showing that the global demand, particularly in high-income countries, for primary commodities linked to deforestation could potentially escalate malaria risk in developing tropical countries.

The value chain underlying the international processing of malaria-implicated commodities is highly unequal. On the one hand, producers of low-value cash crops such as timber, cocoa, or charcoal in malaria-endemic countries are dependent on export revenues for poverty reduction and economic development, and thus enter into an ecological race to the bottom. On the other hand, the lion’s share of value added accrues to high-income countries where such export commodities are manufactured into consumer goods such as furniture and chocolate. In this unequal value chain, ecosystem degradation and malaria risk are borne by low-income producers, but the associated indirect and often long-term cost are not included in their trade revenues^67^. Thus, these low-income countries pay for their cash crop export incomes with a burden from increased malaria risk. Such outsourcing is not new, it also occurs globally for pollution-intensive production, and predominantly from developed to developing countries^68–70^.

It is true that the main importers of malaria-implicated products, the USA, UK, France, Germany, and Japan, provide financial support for malaria control programs, especially in Sub-Saharan Africa. In 2017, global investment for malaria control and prevention was approximately US$3.2 billion, with high-income donors providing 72% of funding (USA 39%, Development Assistance Committee 21%, UK 9%, Bill & Melinda Gates Foundation 2%). However, malaria-endemic countries shouldered 28% of the cost, and the overall funding level is less than half of what is required^2^ to achieve a reduction in malaria morbidity and mortality rates in line with goal 3.3 of the UNSDA^5^.

Rather than relying only on malaria control, the results from this study show the possibility of additional opportunities arising from international supply-chain relationships, addressing the demand structures that lead to export-related deforestation in the first place^71,72^. First, targeting the demand for malaria-implicated products assists in reducing the constant need for malaria control. Second, robust demand-side measures do not interfere with, but complement existing malaria control measures. Third, and perhaps most importantly, demand-side policies can align with other initiatives that focus on forest-related commodities, such as the Eliminating Deforestation from the Cocoa Supply Chain^48^, the Soy Moratorium in Brazil^73^, the G4 Zero-Deforestation Agreement, and the Terms of Adjustment of Conducts^74^, among others^75^. These existing measures can readily be enhanced towards a more comprehensive perspective, mirroring the UNSDA goals^5^, by targeting malaria risk alongside deforestation.

Demand-side measures can be established at several points within the international supply-chain network: (i) by engaging consumers, (ii) by stimulating producer dialog within the intermediate supply chains, (iii) by legislating certification and other producer standards with the help of governments and NGOs, and (iv) through fiscal instruments.

Demand-side measures can be motivated by corporate perceptions of consumers’ attitudes, or in the absence of pro-active engagement by companies, through consumer pressure. A well-known example is consumers pushing companies using palm oil toward ensuring that their products are deforestation-free^76^. As with dolphin-safe tuna, fair-trade chocolate, organic produce or sustainable seafood labelling, an effective way to ensure that products are not malaria-implicated is through certification, based on information about economic, ecological, social and health attributes of the products’ supply chains. Product-specific certification standards can be designed, benefitting especially smallholders, by building a tangible set of criteria that associate the product with sustainable production attributes that avoid environmental degradation and malaria risk, as well as promote the local community. Certification, in turn, can improve smallholders’ organisation, political leverage and land tenure, their access to funds, training and technology, and ultimately enhance their production capacity^77^.

Further upstream in the supply chain, companies can more pro-actively engage in supply-chain governance. Currently, manufacturers, wholesalers and retailers in high-income countries are often far removed from smallholders’ economic situations, and the environmental degradation and malaria risk that these entails. This results in them ignoring the adverse effects caused by the production of the basic commodities they buy. Companies can assume responsibility for their role in the global supply chain, and incorporate into their advertising and sales strategies, the ecological and health limits associated with their inputs. Companies can also use their supply-chain leverage by mandating certain procurement standards and exercising their procurement practices accordingly. Such pro-active engagement will ultimately mitigate companies’ risk of supply shortages, for example due to overexploitation of land, or malaria incidence amongst workers, and improve their long-term productivity. To this end, Ostrom et al.^78^ propose a multilevel-institutions approach that builds on a supply-chain dialog that strongly involves local and regional institutions. The Roundtable for Sustainable Palm Oil (RSPO) shows the effort and commitment of producer and importer countries and companies involved in the trade and processing of oil to adopt sustainable practice for production and commercialization at all levels, across groups of companies^79^. Therefore, improving the dialog among local and regional producers, governments, and sale companies can help to increase productivity without deforestation, thus decreasing malaria risk for local producers.

Governments and NGOs can play an important role in proposing unique strategies and policies for production of basic commodities in endemic countries. These include training courses for local producers about new technologies and cultivation methods for malaria-implicated crops such as cocoa or coffee. In addition, the joint collaboration between government, private companies, NGOs, and society will improve monitoring and commitment of those involved in the trade chain and consumption. Such initiatives exist already for influencing initiatives focusing on interventions for decreasing biodiversity loss in response to overexploitation of commodities^80^, monitoring illegal wildlife trade governed by the Convention on International Trade in Endangered Species of Wild Fauna and Flora (CITES)^81^, and voluntary corporate disclosure of carbon emissions (CDP)^82^. These activities provide high-income, developed countries with an additional opportunity for assisting smallholder producers in malaria-endemic countries in ensuring the legality and certification of their products. In this way, it will be possible to maintain transparency throughout the production process to guarantee the commercialization of origin-certified products^51,76^.

More stringent measures are fiscal instruments such as taxes aimed at stemming overexploitation of natural resources^83^. However, their imposition on international trade may conflict with WTO rules. An alternative means is the distribution of royalties for the ongoing use of natural resources to sustainable-certified smallholder cooperatives, generating a virtuous cycle of improving productivity whilst decreasing overexploitation, deforestation, and malaria risk.

Having identified a number of demand-side measures to reduce malaria risk that address the potential role of international trade, it is important to point out that these must be designed with potentially vulnerable smallholders in mind. The commercial trade of basic commodities is a particularly important and reliable source of cash income for smallholders, who exploit natural vegetation, leading to deforestation and malaria^11,84–86^. For instance, charcoal production is closely linked to deforestation in sub-Saharan Africa. Recognising that the complex interplay between charcoal production, deforestation, forest degradation, land use changes and tenure systems is determined by the charcoal value chain, approaches to reducing deforestation and malaria associated with charcoal production must incorporate innovative technologies that allow growing trees with high energetic content for charcoal production, thus increasing the commercial value of the product and decreasing land pressure^87,88^. Recent initiatives to ensure corporate commitments aimed at reducing the sale of deforestation-implicated commodities have not yielded robust results. Our study therefore provides evidence for governments of malaria endemic countries to undertake action for ensuring health policies and malaria control programs are implemented in collaboration with local communities and other stakeholders, in a global anti-malaria strategy. In general, sharing responsibility, and obtaining commitments and coordinated support from entire product value chains, involving local governments, investors, traders, manufacturers and consumers, will be essential for establishing robust tenure systems, and ensuring smallholders’ livelihood, whilst at the same time reducing deforestation and malaria risk.

## Methods

### Definition and calculation of malaria risk

**Fig. 5:**
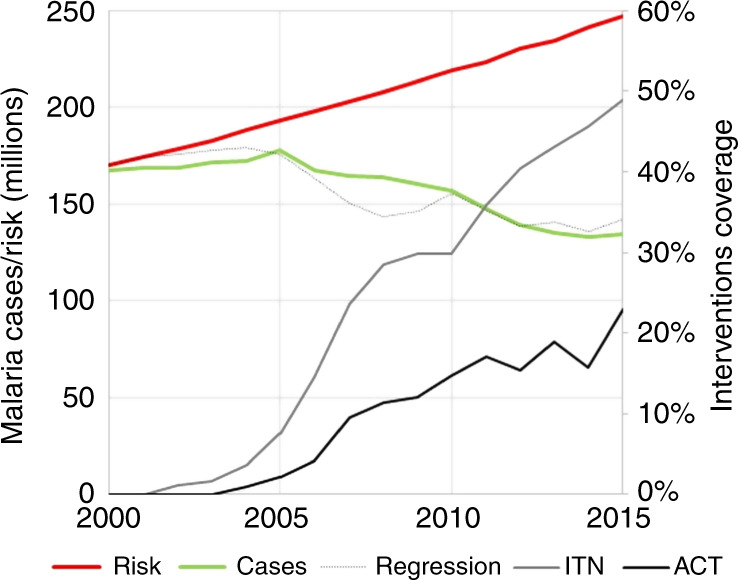
Malaria incidence data over the 2000–2015 time period. Multiple regression (black dotted curve) of malaria incidence data (green) over the 2000–2015 time period, as a function of tree cover loss, the proportion of populations at risk using ITN (grey), and the proportion of populations at risk using ACT (black). Overall malaria risk (red) is determined by evaluating the multiple regression formula for existing tree cover loss, but assuming zero usage of ITN and ACT^64, 89^. Considering the high *R*^2^ of the regression, we attribute the difference between the red and the green curves to deforestation.

We define malaria risk by how many cases there would be in the presence of deforestation but in the absence of any public health intervention. There exists information on the evolution of actual malaria cases over time (green curve in Fig. 5), and this information reflects many factors influencing malaria trends, such as deforestation and interventions, but also migration, temperature and climate change, amongst others. Our approach is simply to take interventions out of this trend. We do this in a three-step procedure: first, we regress actual malaria cases against interventions. Secondly, we take the regression equation and set the intervention variables to zero. Thirdly, this yields the estimated malaria cases in the absence of intervention, i.e. “malaria risk” as we have defined it.

### Multiple regression

To do this, we undertake in the first step a multiple regression that fits the trends of actual malaria cases with a sufficiently high goodness of fit (*R*^2^ = 0.91), and that explicitly includes deforestation and interventions as explanatory variables. We use individual data-tuples for each year in the regression analysis. To this end, we subject current malaria incidence data *I*_*r*_(*t*) in countries *r*, over the 2000–2015 time period^89^ to a multiple regression against (a) cumulative tree cover loss *L* since 2000^64,90^, (b) the proportion *n*(*t*) of the populations using ITN^91,92^, and (c) the proportion *a*(*t*) of populations using ACT^91,92^, as ${\sum}_r {I_r\left( t \right)} = \beta _0 + \beta _L {\sum}_r {L_r\left( t \right) + \beta _nn\left( t \right) + \beta _aa\left( t \right)}$. Although we assume a linear relationship in our regression, the relationship between malaria, tree cover loss and interventions does not necessarily have to be linear. Our approach was to undertake a multiple regression that fits the trends of actual malaria cases with a sufficient *R*^2^, initially for the simplest functional relationship, which is linear. We found *R*^2^ = 0.91. Of course, we could move to polynomial or other functional forms, but the improvement on the overall *R*^2^ would be small, a maximum of 0.09. A different functional form leading to a few 0.01 point of improvement to the *R*^2^ will necessarily have to closely mimic the linear form that achieves *R*^2^ = 0.91. Therefore, we do not expect that an increase of a few 0.01 in the *R*^2^ as a result of a different functional form will change the results in the paper. This formulation implies that factors other than deforestation and intervention—for example forest environment (savannah and cultivation vs closed forest), migration, or temperature are contained in the constant (*β*_0_ = 170.0).

The malaria incidences for the years 2001, 2005 and 2014 were badly represented by an unweighted regression model. To this end, we attempted to fit the incidence curve with a weighted function including interventions and tree cover loss (TCL). This approach is justified because the goal of our regression is to model the influence of deforestation on malaria incidence. Without weighting, a significant and positive regression coefficient for TCL cannot be obtained. The regression formula including weights *ω(t)* as is ${\omega} (t) {\sum}_r \ {{I}}_r{(t)} = \beta _0 + \beta _L \ {\omega} (t) {\sum}_r {L_r\left( t \right) + \beta _n {\omega} (t) n\left( t \right) + \beta _a {\omega}\left( t \right)}{\rm{a}}(t)$, with weights being 17.86 percent for 2001, 42.86 percent for 2005 and 16.07 percent for 2014. The regression coefficients from this approach are *β*_*0*_ = 164.1, *β*_*L*_ = 0.43, *β*_*n*_ = −243.3, and *β*_*a*_ = −72.4. The choice of weights has only a small influence on regression outcomes, and the land cover coefficient is similar to that found by MacDonald and Mordecai (2019), who found a value of 3.3% increase in malaria incidence as a result of 10% increase in forest cover loss.

With the regression’s *R*^2^ = 0.91 (dotted black curve in Fig. 5), we find *β*_0_ = 170.0 ± 1.8 × 10^6^ cases (significant at the 99%-level of confidence), *β*_*L*_ = 0.31 ± 0.05 × 10^6^ cases per million hectares cumulative tree cover loss (99%), *β*_*n*_ = −282 ± 38 × 10^6^ cases per %ITN (99%), and *β*_*a*_ = 143 ± 61 × 10^6^ cases per %ACT (95%).

In the second step, we estimate overall malaria risk as incidence in the presence of deforestation, but in the absence of ITN and ACT interventions by evaluating the parametrized incidence regression formula for *n*(*t*) = a(*t*) = 0∀*t*, that is ${\sum}_r {R_r\left( t \right)} = \beta _0 + \beta _L {\sum}_r {L_r\left( t \right)}$ (*β*_0_ = 170.0), the red solid curve in Fig. 5. This measure of risk reflects that despite interventions, vector abundance increases, and populations remain vulnerable because of continuing deforestation. In order to isolate the portion of malaria risk that is attributable to deforestation (as opposed to factors such as the forest environment), we subtract actual incidences from overall malaria risk: ${\sum}_r {R_{r{\mathrm{,def}}}\left( t \right)} = {\sum}_r {R_r\left( t \right)} - {\sum}_r {I_r\left( t \right)}$. Individual countries’ malaria risk values R_*r*,def_(*t*) are derived then from the global total as R_*r*,def_(*t*) = ∑_*r*_*R*_*r*,def_(*t*) * *I*_*r*_(*t*)/∑_*r*_*I*_*r*_(*t*).

### Basics of input–output analysis

The set of sectoral malaria risk values formed the satellite block (**Q** block) for the MRIO model. A satellite block is a so-called physical account matrix that holds information on physical data (e.g. related to malaria risk), which is further coupled with an economic multi-regional input–output table for quantifying the role of global consumption and international trade in driving malaria risk.

Input–output (IO) analysis was conceived by Nobel Prize Laureate Wassily Leontief in the 1930s and 1940s^93,94^. Today more than 100 statistical agencies around the world regularly publish national IO databases in common formats governed by UN standards^95–97^. At the heart of IO analysis lies a set of matrices describing economic interdependence. The transactions matrix **T** holds elements *T*_*jk*_ representing the supply of commodity *j* for use in industry *k*. Commodities and industries spans the entire range of production, from agriculture, fishing forestry and mining (primary) to manufacturing (secondary) and construction, utilities, trade, hospitality, transport, communications, government administration and other services (tertiary). The final demand matrix **Y** holds elements *Y*_*kl*_ representing the supply of commodity *k* for use by final demand agent *l*. Final demand agents are households, the capital sector, the government and inventories. The value-added matrix **V** holds elements *V*_*ij*_ representing the supply of primary input *i* for use by industry *j*. Primary inputs are wages and salaries, gross operating surplus, taxes and subsidies.

Leontief’s basic accounting identity reads^36^ **1**^**V**^**V**’ + **1**^**T**^**T**’ = **T1**^**T**^ + **Y1**^**Y**^ = **x**,

where the **1** = {1,…,1} vectors are suitable row or column summation operators, **x** is total output, and the prime **‘** denotes transposition. Calling ${\mathbf{A}} = {\mathbf{T}}\widehat {\mathbf{x}}^{{{ - {{1}}}}}$, where the hat **^** symbol denotes vector diagonalization, the accounting identity transforms into ${{{\mathbf{Ax}} \,{\mathbf{+}}\, {\mathbf{Y1}}^{\mathbf{Y}} = {\mathbf{x}} \Leftrightarrow \left( {{\mathbf{I}} - {\mathbf{A}}} \right){\mathbf{x}} = {\mathbf{Y1}}^{\mathbf{Y}} \Leftrightarrow {\mathbf{x}} = \left( {{\mathbf{I}} - {\mathbf{A}}} \right)^{{{ - {\mathbf{1}}}}}{\mathbf{Y1}}^{\mathbf{Y}} = {{:{\rm{L}}}}{\mathbf{Y1}}^{\mathbf{Y}},}}$ where **I** is an identity matrix with *I*_*ij*_ = 1 if *i* = *j* and 0 otherwise, and where ${\mathbf{L}}: = \left( {{\mathbf{I}} - {\mathbf{A}}} \right)^{{{ - {{1}}}}}$ in the first mention only.

This inverse matrix links final demand **Y**1^**Y**^ and total output **x**, and thus incorporates the structure of the entire supply-chain network linking consuming households with producing industries^98^. Because ${\mathbf{A}} = {\mathbf{T}}\widehat {\mathbf{x}}^{{{ - {{1}}}}}$, we find that ${\mathbf{L}}: = ( {{\mathbf{I}} - {\mathbf{T}}[ {\widehat {{\mathbf{T1}}^{\mathbf{T}} \!+ {\mathbf{Y1}}^{\mathbf{Y}}}} ]^{{{ - {{1}}}}}} )^{{{ - {{1}}}}}$, and thus, **L** can be computed from the transactions and final demand matrices published by national statistical agencies. It is this matrix that is interrogated in the extraction of malaria footprints from the global supply-chain network. Table SI 1 shows the export–import malaria risk associated with products-led deforestation, caused by just ten countries (net importers in Fig. 2).

### Construction of satellite account

Values of *R*_*r*,def_(*t*) are then used to construct a satellite account for the MRIO database. To this end, these values must be distributed across economic sectors. We used country-wise FAOSTAT data to estimate the contribution of selected commodities to overall tree cover loss. We identify the following commodities in FAOSTAT as being associated with deforestation: soybeans, oil palm fruit, cattle, sheep, buffalo, timber, wood products (such as furniture), tobacco, cocoa, coffee and cotton. We use the FAOSTAT land-use (hectares) dataset to calculate the year-to-year land-use change for each commodity crop. For livestock commodities, where FAOSTAT data offers only animal head count information, we calculate land-use area by multiplying head counts with estimated stocking rates. The yearly change in land-use was then used to apportion malaria risk between the selected commodities. For each country in the underlying MRIO, these FAOSTAT commodities can be mapped to a unique sector in the corresponding country classification. Hence, by using the FAOSTAT data as a proxy, we were able to assign the tree cover loss data to each country in the Eora MRIO without the need for further manual allocation. This mapping was then used to allocate the country-wise malaria risks to the individual sectors within each country.

### Data sources and statistical analysis

The global Leontief inverse $L_{{{ij}}}^{rs}{\mathrm{,}}$its components $A_{{{ij}}}^{rs}$, as well as data on global final consumption $y_j^{st}$ and total output *x*_*i*_ were taken from the Eora MRIO database^38^. A malaria risk matrix $Q_i^r$ was determined in a three-step procedure: First, we collected data on actual malaria incidence *I*_*r*_(*t*) in countries *r*, over the 2000–2015 time period^2^, and subjected these to a multiple regression against (a) tree cover loss *L*^64,90^, (b) the proportion *n*(*t*) of populations using ITN (insecticide-treated mosquito nets)^89^, and (c) the proportion *a*(*t*) of populations using ACT (artemisinin-based combination therapies)^89^, as ${\sum }_r^{} I_r\left( t \right) = \beta _0 + \beta _L {\sum }_r^{} L_r\left( t \right) + \beta _nn\left( t \right) + \beta _aa\left( t \right)$. Second, we estimate overall malaria risk *R*_*r*_(*t*) as incidence in the presence of deforestation, but in the absence of ITN and ACT interventions by evaluating the parametrized incidence regression formula for *n*(*t*) = a(*t*) = 0∀*t*, that is ${\sum }_r^{} R_r\left( t \right) = \beta _0 + \beta _LL_r\left( t \right)$ (see Fig. 5). Finally, we attribute the difference between overall malaria risk and actual cases to deforestation: ${\sum }_r^{} R_{r{\mathrm{,def}}}\left( t \right) = {\sum }_r^{} R_r\left( t \right) - {\sum }_r^{} I_r\left( t \right)$. Thus, we measure malaria risk using a counterfactual: how many additional cases would there be in the presence of deforestation but in the absence of any public health intervention within each country. This is thus a synthetic unit, but a transparent and effective one for signaling the magnitude of the public health threat exerted by export-induced deforestation. This measure of risk reflects that despite interventions, vector abundance increases, and populations remain vulnerable because of continuing deforestation, insecticide resistance, treatment allergies, discontinuities in control interventions, or inability to pay for imported intervention technology^71^. Third, we used tree cover loss and commodity production^99–101^ data to allocate country totals *R*_*r*,def_(*t*) of malaria risk across deforestation-implicated commodities. This way, we finally arrived at a malaria risk matrix $Q_i^r$, satisfying ${\sum }_i^{} Q_i^r = R_{r{\mathrm{,def}}}\left( t \right)$, individually for every year and country.

### Modeling approach

Whilst in 2015, about 135 million cases of malaria were recorded globally, we estimate that an additional 110 million people remain at risk of contracting malaria due to continued deforestation (Fig. 5). Here, ‘at risk’ means either actual cases requiring costly treatment (for example: purchase of medicines such as artemisinin-based combination therapies) or vulnerability requiring costly intervention (e.g. distribution of insecticide-treated mosquito nets). Correspondingly, “malaria risk” means the number of potential malaria cases in the presence of deforestation but in the absence of public health interventions.

### Reporting summary

Further information on research design is available in the Nature Research Reporting Summary linked to this article.

## Supplementary information

Supplementary Information

Reporting Summary

## Data Availability

Codes and data for figures and tables are available on Zenodo repository (10.5281/zenodo.4536029). Data on global final consumption and total output were taken from the Eora MRIO database that consists of a multi-region input–output table (MRIO) model providing a time series of high-resolution IO tables with matching environmental and social satellite accounts for 190 countries (https://worldmrio.com)^102^. Global tree cover loss data were collected from Global Forest Change^64^. Data on malaria commodities were obtained from MAP—Malaria Atlas Project^89^, (https://map.ox.ac.uk). Commodity data were obtained from FAO—Food and Agriculture Organization^103^ (http://www.fao.org/faostat/en). All malaria cases data were collected from World Health Organization (WHO - https://www.who.int/malaria/publications/world_malaria_report). The underlying MRIO data are associated with measurement errors^102^, uncertainty and reliability in the Eora MRIO tables (https://worldmrio.com/documentation/), however due to their stochastic nature these tend to cancel out when performing the Leontief analysis (Basics of input–output analysis). Aggregate MRIO-based measures such as reported in this work are typically characterized by uncertainties of around 10%, and existing assessments show that results obtained from different MRIO databases agree well^104^.
