# Author Correction: Global consumption and international trade in deforestation-associated commodities could influence malaria risk

**DOI:** 10.1038/s41467-021-23385-5

**Published:** 2021-06-11

**Authors:** Leonardo Suveges Moreira Chaves, Jacob Fry, Arunima Malik, Arne Geschke, Maria Anice Mureb Sallum, Manfred Lenzen

**Affiliations:** 1 Departamento de Epidemiologia, Faculdade de Saúde Pública, Universidade de São Paulo, São Paulo, SP Brazil; 2 ISA, School of Physics A28, The University of Sydney, Sydney, NSW 2006 Australia; 3 Discipline of Accounting, The University of Sydney Business School, The University of Sydney, Sydney, NSW 2006 Australia

**Keywords:** Ecological epidemiology, Environmental economics, Risk factors

Correction to: *Nature Communications* 10.1038/s41467-020-14954-1, published online 09 March 2020.

The original version of the Article omitted details from the Methods section explaining the weighting scheme in the regression analysis. It was not clear in the original Methods text that the authors weighted some years more than others in the analysis.

To more thoroughly explain the methods, the new version of the Article has the following paragraph between the original first and second paragraphs of the “Multiple regression” section of the Methods:

“The malaria incidences for the years 2001, 2005 and 2014 were poorly represented by an unweighted regression model. To this end, we attempted to fit the incidence curve with a weighted function including interventions and tree cover loss (TCL). This approach is justified because the goal of our regression is to model the influence of deforestation on malaria incidence. Without weighting, a significant and positive regression coefficient for TCL cannot be obtained. The regression formula including weights *w(t)* as is *w(t)*∑_*r*_*I*_*r*_(*t*) = *β*_0_ + *β*_*L*_*w*(*t*)∑_*r*_*L*_*r*_(*t*) + *β*_*n*_*w*(*t*)*n*(*t*) + *β*_*a*_*w*(*t*)*a*(*t*), with weights being 17.86 percent for 2001, 42.86 percent for 2005 and 16.07 percent for 2014. The regression coefficients from this approach are *β*_0_ = 164.1, *β*_*L*_ = 0.43, *β*_*n*_ = −243.3, and *β*_*a*_ = −72.4. The choice of weights has only a small influence on regression outcomes, and the land cover coefficient is similar to that found by MacDonald and Mordecai (2019), who found a value of 3.3% increase in malaria incidence as a result of 10% increase in forest cover loss.”

In the data file originally provided on Zenodo, there were duplicated rows for years 2001, 2005 and 2014 which were weighted more heavily, but these rows were hidden. For the sake of transparency, the dataset has also been updated with the rows unhidden; the data file can be accessed on Zenodo (10.5281/zenodo.4536029). These errors have been corrected in the original PDF and HTML versions of the article.

