# Supplementary material for: Global consumption and international trade in deforestation-associated commodities could influence malaria risk
Source: Nat Commun. 2020 Mar 9;11:1258. doi: 10.1038/s41467-020-14954-1 (PMC7062889; doi:10.1038/s41467-020-14954-1)
Supplement: Supplementary file 1 — Supplementary Information [file 41467_2020_14954_MOESM1_ESM.pdf]

Global consumption and international trade in deforestation-associated commodities could influence malaria risk

Leonardo Suveges Moreira Chaves[1,2*], Jacob Fry[2], Arunima Malik[2,3], Arne Geschke[2], Maria Anice Mureb Sallum[1&], Manfred Lenzen[2&*]

[1] Departamento de Epidemiologia, Faculdade de Saúde Pública, Universidade de São Paulo, Av. Dr. Arnaldo, 715. CEP – 01246-904. São Paulo – SP. Brazil; phone: 55 (11) 3061-7951, fax 55 (11) 3061-7926

[2] ISA, School of Physics A28, The University of Sydney, NSW 2006, Australia

[3] Discipline of Accounting, The University of Sydney Business School, The University of Sydney, NSW, 2006, Australia.

[*] Corresponding authors. E-mail: leonardosuveges@usp.br. E-mail: manfred.lenzen@sydney.edu.au

[&] Senior authors

Table of contents

Supplementary Note 1: Deforestation and malaria risk 2
Table SI 1. Export-import malaria risk embodied in products-led deforestation. 7
References 9

Supplementary Note 1: Deforestation and malaria risk

Possibly the earliest record of the environment playing an important role in the occurrence of malaria stems from Lower Mesopotamia (modern-day Iraq) in approximately 4000 BP, when the disease was associated with the presence of swamps. Sumerian records mention constant occurrence of deadly epidemic fevers, likely malaria, a disease that was earlier associated with "bad air" (mal'aria) from the swamps present in the region. There are also accounts of malaria outbreaks in classical Greece (approximately 2500 BP) coinciding with anthropogenic landscape changes for agriculture, indicating a potential correlation between deforestation and the incidence of malaria[1].

Several studies have demonstrated a strong association between deforestation and an increase in malaria incidence[2-12], with the former being driven by the expansion of agriculture, road construction, mining, logging for economic growth. Within the concept of deforestation-malaria link, malaria transmission is determined by a multitude of factors, including the local environment, climate, mosquito vector ecology, *Plasmodium* reservoir, human behaviour, agricultural development, and commodities production. Deforestation has been associated with ecological changes in the environment, which can alter the community of mosquitoes present in tropical forest, favouring those species that transmit human pathogens[13,14]. For example, following significant deforestation events, the increased abundance of the *Anopheles* and *Nyssorhynchus* vectors of *Plasmodium* is known to have caused widespread malaria epidemics[15-17]. Therefore, deforestation, selective logging, wildfires and land use change represent the main pathway for increased malaria in receptive landscapes[6,18,19]. Recent articles[2,3 6,9,11,12,20] reinforce and give support to the link between deforestation and malaria, showing magnitude and direction for the relationship,

indicating that small patches of forest cleared for human land use, lead to an increase in malaria risk. In Brazil, the expansion of cattle ranching for beef production is a major cause of deforestation in the Amazon[21], where 99% of malaria cases occur[22]. Soybean production has also been shown to displace cattle ranching to other regions[23], thus promoting additional landscape changes[24]. The continuous process of changes in natural forest environments increases the potential for emergence of malaria outbreaks into new areas that are susceptible to the presence of mosquito vector species. Approximately one third of deforestation is associated with exports of natural resources and commodities, such as timber, soybean and beef[25].

A highly detailed multi-regional input-output database and high-performance computation was used to investigate an association among malaria incidence in developing countries, global consumption and international trade. Specifically, we investigated how global consumption and the international trade of commodities expedite the global demand for timber, agricultural products, minerals and food, and thus push new deforestation fronts, and increase malaria risk. In this interdisciplinary study, economic drivers of landscape change were addressed as important factors in explaining an increase in malaria risk. Malaria risk is herein defined as incidence in the presence of deforestation, but in the absence of interventions such as insecticide-treated mosquito nets and artemisinin-based combination therapies. Thus, our malaria risk measure reflects the chance of vectors' infectious bites, considering the biological and ecological transmission processes enhanced by landscape change.

In a recent study, Garg[8] demonstrated that after controlling several malaria covariables, deforestation is associated with an increase in malaria in Indonesia. Although Garg

conducted his study in Indonesia as a representative of malaria endemic countries, his findings are largely applied to other tropical and subtropical malaria endemic countries.

The malaria model proposed by Ross and Macdonald[26] includes metrics for measuring transmission, and key determinants in the transmission cycle. The central assumption in Ross & Macdonald model is the homogeneous transmission in a well-mixed population. Considering that malaria dynamics are nonlinear (heterogeneous) with focal determinants that are largely unexplored, Smith et al.[5] proposed to recast malaria models to include multiple determinants of malaria landscapes, which range locally, regionally and globally.

Heterogeneities in the malaria transmission cycle are determined by variations in factors of the three major components: entomological components, parasites components, and human components, which interact in a dynamic environment[27]. The entomological components of malaria vary depending on characteristics of mosquito ecology, heterogeneous blood-feeding and biting behavior, mosquito feeding cycle, mosquito genetics, spatial-temporal mosquito population fluctuations, abiotic and biotic factors of the environment that regulate mosquito populations, aquatic mosquito ecology, oviposition behavior and mosquito movement[5]. The socioeconomic context of malaria transmission is associated with the degree of human exposure to mosquito bites, human mobility, human behavior, poverty, and other factors of human ecology that enable *Plasmodium* propagation in a population[27].

Considering the vital importance of field studies in malariology, Baird[28] defended that the effectivity of malaria control interventions depends on field studies conducted to obtain data of mosquito ecology and behavior, mosquito and vertebrate host movement, human ecology, and spatial heterogeneity in complex epidemiological landscapes. In addition, knowledge of baseline epidemiological parameters of transmission, including intervention

coverage, vector ecology and the way those factors determine the dynamics of transmission will be necessary to predict the outcomes of malaria interventions[7].

Regarding deforestation and malaria risk, recent studies provided evidence of causal association of primary forest cover loss and an increase in malaria incidence in Indonesia[8], and in children in Nigeria[10]. It is worth noting that Garg´s findings supported that malaria is an ecological response of deforestation and land-use change, and that malaria control interventions and human migration cannot explain the effect of forest cover loss and increased malaria incidence. Equally important, the author state that: *"Ecosystem degradation poses a fundamental threat to lives and livelihoods, especially in the poorest parts of the world. In this paper, I provide evidence on the effect of sustained primary forest cover on reducing malarial incidence in Indonesia. Specifically, a one standard deviation increases in primary forest cover reducing the probability of malarial outbreak by over 13% with overall primary forest cover generating malaria-related morbidity-reducing benefits of almost $100 million. The effects are specific to malaria and specific to primary forests implying an ecological mechanism underpinning the relationship between forest cover and malaria. Importantly, these estimates present the short-run benefits of sustained forest cover. Future work should examine how more permanent and sustained changes in ecosystems affect disease ecologies and incidence of infectious diseases to uncover a key missing ingredient in willingness to pay for ecosystem preservation (Fenichel and Abbott, 2014). Together, this body of research should not only inform program designs for payments for ecosystem services, but also provide new evidence on immediate and localized benefits of ecosystems that have been previously understudied."* In

Nigeria, malaria incidence in children was found to increase in the first and second year after deforestation, thus returning to previous level in the third year after forest cover loss[10].

We found that global malaria implicated commodities include timber, wood products (such as furniture), tobacco, cocoa, coffee and cotton. Thus, policies aiming at sustainable development, biodiversity protection, carbon sequestration, fair wages and poverty reduction, can present ways for decreasing malaria incidence in endemic countries. Results of the study can be used for designing new demand-side approaches to mitigating malaria incidence by focusing on regulating malaria-implicated global supply chains. These approaches can effectively complement agroforest management and malaria control interventions such as insecticide-treated mosquito nets and artemisinin combination therapies. Demand-side initiatives such as product labeling and certification, supply-chain dialogue and green procurement standards have been successful in addressing trade-related global problems such as deforestation, threats to species, and child labour[29,30], and can therefore be directed at reducing malaria incidence as well.

Table SI 1. Export-import malaria risk embodied in products-led deforestation.

	Exports				Imports				
	Primary		Secondary		Primary		Secondary		
**Countries**	**Malaria Risk (‘000)**	**Per-Capita × 1000**	**Malaria Risk (‘000)**	**Per-Capita × 1000**	**Malaria Risk (‘000)**	**Per-Consumer × 1000**	**Malaria Risk (‘000)**	**Per-Consumer × 1000**	**Population (millions) (2015)[1]**
Nigeria	3762	20.8	308	1.7	7	0.0	12	0.1	181.2
Tanzania	2137	39.6	804	14.9	16	0.3	8	0.2	53.9
Cameroon	1925	84.4	474	20.8	6	0.3	7	0.3	22.8
Uganda	1297	32.3	484	12.1	16	0.4	11	0.3	40.1
DR Congo	1313	17.2	270	3.5	151	2.0	116	1.5	76.2
India	1034	0.8	679	0.5	704	0.5	126	0.1	1309.1
Zambia	636	39.5	316	19.6	12	0.7	8	0.5	16.1
Myanmar	91	1.7	23	0.4	0	0.0	0	0.0	52.4
CAR	271	60.2	53	11.8	2	0.4	2	0.4	4.5
Burundi	139	13.6	46	4.5	4	0.4	0	0.0	10.2
Belgium	0	0.0	173	15.3	361	32.0	142	12.6	11.3
Netherlands	0	0.0	322	19.1	581	34.4	211	12.5	16.9
Spain	0	0.0	89	1.9	466	10.0	205	4.4	46.4

	Exports				Imports				
	Primary		Secondary		Primary		Secondary		
**Countries**	**Malaria Risk (‘000)**	**Per-Capita × 1000**	**Malaria Risk (‘000)**	**Per-Capita × 1000**	**Malaria Risk (‘000)**	**Per-Consumer × 1000**	**Malaria Risk (‘000)**	**Per-Consumer × 1000**	**Population (millions) (2015)[1]**
Italy	0	0.0	149	2.4	595	9.8	251	4.1	60.7
France	0	0.0	85	1.3	463	6.9	556	8.3	66.6
UK	0	0.0	90	1.4	815	12.5	303	4.6	65.1
China	0	0.0	127	0.1	1715	1.3	260	0.2	1371.2
Japan	0	0.0	45	0.4	986	7.8	143	1.1	127.1
USA	0	0.0	31	0.1	769	2.4	414	1.3	320.7
Germany	0	0.0	518	6.3	1509	18.5	460	5.6	81.7

[1] World Development Indicators, The World Bank

References

1. Bruce-Chwatt, L. J. History of malaria from prehistory to eradication. Malaria: Principles and Practice of Malariology. Wernsdorfer WH, McGregor I. Edinburgh: Churchill Livingstone **1**, 1–59 (1988).
2. Patz, J. A., Graczyk, T. K., Geller, N. & Vittor, A. Y. Effects of environmental change on emerging parasitic diseases. *International Journal for Parasitology* **30**, 1395–405 (2000).
3. Patz, J. A. et al. Unhealthy landscapes: policy recommendations on land use change and infectious disease emergence. *Environmental health perspectives* **112**, 1092–1098 (2004).
4. Foley, J. A. et al. Global consequences of land use. *Science* **309**, 570–574 (2005).
5. Smith, D. L. et al. Recasting the theory of mosquito-borne pathogen transmission dynamics and control. *Transactions of the Royal Society of Tropical Medicine and Hygiene* **108**, 185–197 (2014).
6. Hahn, M. B. et al. Influence of deforestation, logging, and fire on malaria in the Brazilian Amazon. *PLOS ONE* **9**, e85725 (2014).
7. Brady, O. J. et al. Vectorial capacity and vector control: reconsidering sensitivity to parameters for malaria elimination. *Transactions of the Royal Society of Tropical Medicine and Hygiene* **110**, 107–117 (2016).
8. Garg, T. Ecosystems and human health: The local benefits of forest cover in Indonesia. *Journal of Environmental Economics and Management* **98**, 102271 (2019).

9. Austin, K., Bellinger, M. & Rana, P. Anthropogenic forest loss and malaria prevalence: a comparative examination of the causes and disease consequences of deforestation in developing nations. *Aims Environmental Science* **4**, 217–231 (2017).

10. Berazneva, J. & Byker, T. S. Does Forest Loss Increase Human Disease? Evidence from Nigeria. *American Economic Review* **107**, 516–521 (2017).

11. Brock, P. M. et al. Predictive analysis across spatial scales links zoonotic malaria to deforestation. *Proceedings of the Royal Society B* **286**, 20182351 (2019).

12. Chaves, L. S. M., Conn, J. E., López, R. V. M. & Sallum, M. A. M. Abundance of impacted forest patches less than 5 km^2 is a key driver of the incidence of malaria in Amazonian Brazil. *Scientific Reports* **8**, 7077 (2018).

13. Vasconcelos, P. F. C., et al. An Epidemic of Sylvatic Yellow Fever in the Southeast Region of Maranhao State, Brazil, 1993–1994: Epidemiologic and Entomologic Findings. *American Journal of Tropical Medicine and Hygiene* **57**, 132–7 (1997).

14. Galactionova, K., Smith, T. A., de Savigny, D. & Penny, M. A. State of inequality in malaria intervention coverage in sub-Saharan African countries. *BMC Medicine* **15** (1), 185 (2017).

15. Ferreira, M. U. & Castro, M. C. Challenges for malaria elimination in Brazil. *Malaria Journal* **15**, 284 (2016).

16. Walsh, J. F., Molyneux, D. H. & Birley, M. H. Deforestation: effects on vector-borne disease. *Parasitology* 106 Suppl: S55–75 (1993).

17. Camargo, L. M. A. et al. Unstable Hypoendemic Malaria in Rondonia (Western Amazon Region, Brazil): Epidemic Outbreaks and Work-Associated Incidence in an

Agro-Industrial Rural Settlement. *American Journal of Tropical Medicine and Hygiene* **51**, 16–25 (1994).

18. Afrane, Y. A. et al. Deforestation and vectorial capacity of *Anopheles gambiae* Giles mosquitoes in malaria transmission, Kenya. *Emerging Infectious Diseases* **14**, 1533–1538 (2008).
19. Sinka, M. E. et al. The dominant *Anopheles* vectors of human malaria in the Asia-Pacific region: occurrence data, distribution maps and bionomic précis. *Parasites & Vectors* **4**, 89 (2011).
20. Norris D. E. Mosquito-borne diseases as a consequence of land use change. *EcoHealth*; **1**, 19–24 (2004).
21. Cederberg, C. et al.. Including carbon emissions from deforestation in the carbon footprint of Brazilian beef. *ACS Publications* (2011).
22. de Pina-Costa, A. et al. Malaria in Brazil: what happens outside the Amazonian endemic region. *Memorias do Instituto Oswaldo Cruz* **109**, 618–33 (2014).
23. Lapola, D. M. et al. Indirect land-use changes can overcome carbon savings from biofuels in Brazil. *Proceedings of the National Academy of Sciences of the United States of America* **107**, 3388–3393 (2010).
24. Arima, E. Y., Richards, P., Walker, R. & Caldas, M. M. Statistical confirmation of indirect land use change in the Brazilian Amazon. *Environmental Research Letters* **6**, 024010 (2011).
25. Richards, P. D., Walker, R. T. & Arima, E. Y. Spatially complex land change: The Indirect effect of Brazil's agricultural sector on land use in Amazonia. *Global Environmental Change* **29**, 1–9 (2014).

26. Smith, D. L. et al. Ross, Macdonald, and a Theory for the Dynamics and Control of Mosquito-Transmitted Pathogens. *PLOS Pathogens*. **8**, e1002588 (2012).

27. Cohen, J. M. et al. Mapping multiple components of malaria risk for improved targeting of elimination interventions. *Malaria Journal* **16**, 459 (2017).

28. Baird, K. J. Malaria control by commodities without practical malariology. *BMC Public Health* **17**, 590 (2017).

29. Lenzen, M. et al. International trade drives biodiversity threats in developing nations. *Nature* **486**, 109–12 (2012).

30. Lenzo, P., Traverso, M., Salomone, R. & Ioppolo, G. Social Life Cycle Assessment in the Textile Sector: An Italian Case Study. *Sustainability* **9**, 2092 (2017).
