# Supplementary material for: Global consumption and international trade in deforestation-associated commodities could influence malaria risk
Source: Nat Commun. 2020 Mar 9;11:1258. doi: 10.1038/s41467-020-14954-1 (PMC7062889; doi:10.1038/s41467-020-14954-1)
Supplement: Supplementary file 2 — Reporting Summary [file 41467_2020_14954_MOESM2_ESM.pdf]

nature research

Corresponding author(s): Leonardo Suveges Moreira Chaves, leonardosuveges@usp.br

Last updated by author(s): Jan 30, 2020

# Reporting Summary

Nature Research wishes to improve the reproducibility of the work that we publish. This form provides structure for consistency and transparency in reporting. For further information on Nature Research policies, see Authors & Referees and the Editorial Policy Checklist .

## Statistics

For all statistical analyses, confirm that the following items are present in the figure legend, table legend, main text, or Methods section.

| n/a | Confirmed | |
|---|---|---|
| x | | The exact sample size (*n*) for each experimental group/condition, given as a discrete number and unit of measurement |
| | x | A statement on whether measurements were taken from distinct samples or whether the same sample was measured repeatedly |
| | x | The statistical test(s) used AND whether they are one- or two-sided<br>*Only common tests should be described solely by name; describe more complex techniques in the Methods section.* |
| | x | A description of all covariates tested |
| | x | A description of any assumptions or corrections, such as tests of normality and adjustment for multiple comparisons |
| | x | A full description of the statistical parameters including central tendency (e.g. means) or other basic estimates (e.g. regression coefficient) AND variation (e.g. standard deviation) or associated estimates of uncertainty (e.g. confidence intervals) |
| | x | For null hypothesis testing, the test statistic (e.g. $F$, $t$, $r$) with confidence intervals, effect sizes, degrees of freedom and $P$ value noted<br>*Give P values as exact values whenever suitable.* |
| x | | For Bayesian analysis, information on the choice of priors and Markov chain Monte Carlo settings |
| | x | For hierarchical and complex designs, identification of the appropriate level for tests and full reporting of outcomes |
| x | | Estimates of effect sizes (e.g. Cohen's *d*, Pearson's *r*), indicating how they were calculated |

*Our web collection on statistics for biologists contains articles on many of the points above.*

## Software and code

Policy information about availability of computer code

| | |
|---|---|
| Data collection | Multi-regional input-output tables used for this study were obtained from Lenzen et al. 2013. These tables are available open-source for research purposes here: <worldmrio.com>. Data on malaria cases were obtained by searching the web using <who.int/malaria/data/en/> |
| Data analysis | Data were integrated and harmonised on High Performance Computers at the School of Physics, The University of Sydney. Analysis was undertaken by compiling a range of MATLAB routines on Matlab R2016b. |

For manuscripts utilizing custom algorithms or software that are central to the research but not yet described in published literature, software must be made available to editors/reviewers. We strongly encourage code deposition in a community repository (e.g. GitHub). See the Nature Research guidelines for submitting code & software for further information.

## Data

Policy information about availability of data

All manuscripts must include a data availability statement. This statement should provide the following information, where applicable:

- Accession codes, unique identifiers, or web links for publicly available datasets
- A list of figures that have associated raw data
- A description of any restrictions on data availability

Codes and data for figures and tables are available on Zenodo repository. Available in: zenodo.org/record/3630653#.XjHMpjJKjIU

# Field-specific reporting

Please select the one below that is the best fit for your research. If you are not sure, read the appropriate sections before making your selection.

- [ ] Life sciences
- [ ] Behavioural & social sciences
- [x] Ecological, evolutionary & environmental sciences

# Ecological, evolutionary & environmental sciences study design

All studies must disclose on these points even when the disclosure is negative.

| | |
|---|---|
| Study description | We present for the first-time evidence of the link between malaria risk in developing countries and developed-world consumption. In our comprehensive global assessment for 189 countries over a time-period from 2000 - 2015, we show how international trade facilitates this link. We demonstrate the prominent role of the global trade network and consumption in high-income countries as drivers of malaria risk, by spurring global market demand for basic commodities linked to deforestation in developing tropical countries. |
| Research sample | We collected data on actual malaria incidence (World Health Organisation database) over the 2000-2015 time period from: Angola, Burundi, Cameroon, Central African Republic, Congo, Democratic Republic of the Congo, Gabon, Kenya, Nigeria, Rwanda, Uganda, United Republic of Tanzania, Zambia, Brazil, Colombia, Peru, Venezuela (Bolivarian Republic of), Bangladesh, India, Indonesia, Myanmar, Thailand, Cambodia, Lao People's Democratic Republic, Malaysia and Viet Nam. Subjected these to a multiple regression against a) tree cover loss, b) the proportion of populations using ITN, c) the proportion of populations using ACT. Global tree cover loss was collected from <http://earthenginepartners.appspot.com/science-2013-global-forest> (accessed March 13, 2018). Commodity data was obtained from FAO - Food and Agriculture Organization <http://www.fao.org/faostat/en/> (accessed March, 2018). |
| Sampling strategy | We obtained this evidence through the application of Multi-Region Input-Output (MRIO) analysis, a technique that has recently contributed high-profile publications in Nature (Lenzen et al. 2012), PNAS (Wiedmann et al. 2015), and the Lancet Planetary Health (Malik et al. 2018). We employ a global multi-regional input-output (MRIO) database to link malaria risk in developing countries with the consumption of commodities in developed countries. Global tree cover loss and commodity production datasets were used to allocate country-level malaria risk across deforestation-implicated commodities. In order to connect malaria risk with global consumption we apply Leontief's method to interrogate a global multi-region input-output (MRIO) database, tracing commodities that were initially obtained as a result of deforestation in tropical forests, then transformed throughout a complex network of international processing chains, and finally delivered to their ultimate destinations in developed-country households. |
| Data collection | Data on global final consumption and total output were taken from the Eora MRIO database, "Eora global supply chain database consists of a multi-region input-output table (MRIO) model that provides a time series of high-resolution IO tables with matching environmental and social satellite accounts for 190 countries" <https://worldmrio.com/>. Data on malaria incidence were obtained from World Health Organisation. Global tree cover loss was collected from <http://earthenginepartners.appspot.com/science-2013-global-forest> (accessed March 13, 2018). Data on malaria commodities were obtained from MAP - Malaria Atlas Project, <https://map.ox.ac.uk> (accessed March 13, 2018). Commodity data were obtained from FAO - Food and Agriculture Organization <http://www.fao.org/faostat/en/> (accessed March, 2018). |
| Timing and spatial scale | Data were collected from over the 2000 until 2015, yearly. |
| Data exclusions | Data on Indoor Resildual Spraying for mosquito vector control was tested with no significant results. Because of this IRS data was excluded for the multiple regression analyses. Considering the results of network analyses showing that just over 10% of malaria risk is caused by10 countries, to construct the net importers in Fig. 2 we used data from them, excluding 179 remaining countries. |
| Reproducibility | Using high-performance computation, we scanned more than a billion international supply chain routes to uncover the contribution of global trade in driving malaria risk in developing countries. |
| Randomization | Randomization is not relevant because we analysed a network of 189 countries and a billion international supply chain routes. |
| Blinding | Blinding is not relevant because we analysed a network of 189 countries and a billion international supply chain routes. |

Did the study involve field work? ☐ Yes ☒ No

# Reporting for specific materials, systems and methods

We require information from authors about some types of materials, experimental systems and methods used in many studies. Here, indicate whether each material, system or method listed is relevant to your study. If you are not sure if a list item applies to your research, read the appropriate section before selecting a response.

## Materials & experimental systems

| n/a | Involved in the study |
|---|---|
| ☒ | ☐ Antibodies |
| ☒ | ☐ Eukaryotic cell lines |
| ☒ | ☐ Palaeontology |
| ☒ | ☐ Animals and other organisms |
| ☒ | ☐ Human research participants |
| ☒ | ☐ Clinical data |

## Methods

| n/a | Involved in the study |
|---|---|
| ☒ | ☐ ChIP-seq |
| ☒ | ☐ Flow cytometry |
| ☒ | ☐ MRI-based neuroimaging |
